# Supplementary material for: Resolving the full spectrum of human genome variation using Linked-Reads
Source: Genome Res. 2019 Apr;29(4):635–45. doi: 10.1101/gr.234443.118 (PMC6442396; doi:10.1101/gr.234443.118)
Supplement: Supplemental Material [file supp_gr.234443.118_Supplemental_longranger-2.2.zip › longranger-2.2.tar.bz2!/lr2.2-public/lib/stan/cmdstan/stan/doc/api/html/context__factory_8hpp_source.html]

Stan: src/stan/common/context\_factory.hpp Source File


|  |  |
| --- | --- |
|  | Stan  2.5.0  probability, sampling & optimization |


- Main Page
- Related Pages
- Namespaces
- Classes
- Files

- File List
- File Members

All Classes Namespaces Files Functions Variables Typedefs Enumerations Enumerator Friends Macros Pages

- src
- stan
- common

context\_factory.hpp

Go to the documentation of this file.

1 #ifndef STAN\_\_COMMON\_\_CONTEXT\_FACTORY\_HPP

2 #define STAN\_\_COMMON\_\_CONTEXT\_FACTORY\_HPP

3

4 #include <fstream>

5 #include <stan/io/dump.hpp>

6

7 namespace stan {

8  namespace common {

9

10  template <typename VARCON>

11  class var\_context\_factory {

12  public:

13  var\_context\_factory() {}

14  virtual VARCON operator()(const std::string source) = 0;

15  typedef VARCON var\_context\_t;

16  };

17

18  // FIXME: Move to CmdStan

19  class dump\_factory: public var\_context\_factory<stan::io::dump> {

20  public:

21  stan::io::dump operator()(const std::string source) {

22  std::fstream source\_stream(source.c\_str(),

23  std::fstream::in);

24

25  if (source\_stream.fail()) {

26  std::string message("dump\_factory Error: the file " + source + " does not exist.");

27  throw std::runtime\_error(message);

28  }

29

30  stan::io::dump dump(source\_stream);

31  source\_stream.close();

32

33  return dump;

34  }

35  };

36

37  }

38 }

39

40 #endif

stan::common::dump\_factory

**Definition:** context\_factory.hpp:19

stan::common::dump\_factory::operator()

stan::io::dump operator()(const std::string source)

**Definition:** context\_factory.hpp:21

stan::common::var\_context\_factory::var\_context\_factory

var\_context\_factory()

**Definition:** context\_factory.hpp:13

stan::common::var\_context\_factory::operator()

virtual VARCON operator()(const std::string source)=0

stan::common::var\_context\_factory::var\_context\_t

VARCON var\_context\_t

**Definition:** context\_factory.hpp:15

stan::common::var\_context\_factory

**Definition:** context\_factory.hpp:11

dump.hpp

stan::io::dump

Represents named arrays with dimensions.

**Definition:** dump.hpp:902


---

[ Stan Home Page ]

*© 2011–2014,
Stan Development Team.*
